# Heat therapy for primary dysmenorrhea: a systematic review and meta-analysis

**DOI:** 10.3389/fmed.2025.1730505

**Published:** 2026-01-23

**Authors:** Dongni Yuan, Yunyu Liu, Ziyi Chen, Zhuoya Hu, Xingxian Li, Wanyi Zhang, Kexin Mao, Wenbin Ma, Lei Lan

**Affiliations:** 1 School of Acupuncture and Tuina, Chengdu University of Traditional Chinese Medicine, Chengdu, Sichuan, China; 2 Clinical Medical College, Chengdu University of Traditional Chinese Medicine, Chengdu, Sichuan, China; 3 School of Health and Rehabilitation, Chengdu University of Traditional Chinese Medicine, Chengdu, Sichuan, China

**Keywords:** primary dysmenorrhea, heat therapy, pain, NSAIDs, meta-analysis

## Abstract

**Aim:**

Primary dysmenorrhea is highly prevalent and often suboptimally managed, as non-steroidal anti-inflammatory drugs (NSAIDs) fail to provide analgesia in 18% of women. This review therefore aims to evaluate the efficacy and safety of heat therapy—a widely used self-care method—for both preventing and acutely treating primary dysmenorrhea.

**Methods:**

We searched seven databases (CENTRAL, PubMed, Web of Science, EMBASE, CNKI, VIP, Wanfang) from inception to October 28, 2024 and updated to August 03, 2025. Pairs of reviewers independently screened records, extracted data, and assessed risk of bias using a modified Cochrane RoB 1.0 tool. Random-effects meta-analyses were performed for pain intensity (converted to 10-cm VAS) and adverse events. Evidence certainty was graded via GRADE (Grading of Recommendations, Assessment, Development, and Evaluations).

**Results:**

We screened 2,733 citations and included 57 RCTs (involving 5,359 female participants). When compared with no treatment, heat therapy may reduce pain intensity to a greater extent after 3 months (25 RCTs, 2,393 females, WMD −1.85 cm, 95% CI −2.29 to −1.41 cm, RD 21%); it may lead to a greater reduction within 24 h of treatment (3 RCTs, 248 females; WMD −3.52 cm, 95% CI −5.01 to −2.02 cm, RD 45%). When compared to NSAIDs, heat therapy may provide comparable or slightly superior pain relief after 3 months of treatment (22 RCTs, 1,938 females, WMD −1.10 cm, 95% CI −1.51 to −0.70 cm, RD 4%), or within 24 h of treatment (2 RCTs, 167 females, WMD −1.50 cm, 95% CI −2.86 to −0.15 cm, RD 16%). For the safety assessment, heat therapy probably reduced the risk of adverse effects compared with NSAIDs (8 RCTs, 728 females, RR 0.30, 95% CI 0.15–0.59).

**Conclusions:**

Compared to no treatment, heat therapy is likely to reduce pain intensity both during prophylaxis and acute episodes. When compared to NSAIDs, heat therapy may achieve comparable analgesic efficacy while exhibiting a superior safety profile.

**Systematic review registration:**

https://www.crd.york.ac.uk/PROSPERO/view/CRD420251050944, identifier CRD420251050944.

## 1 Introduction

Primary dysmenorrhea is a pervasive yet frequently overlooked public health issue, affecting up to 90% of reproductive-aged women worldwide (1). It is defined as painful menstrual cramps in the absence of pelvic pathology (2). The repercussions are substantial, with severe symptoms leading to activity restriction and absenteeism from work or school in up to 15% of affected women, underscoring its considerable socioeconomic burden (3, 4).

Research indicates that women with dysmenorrhea have elevated levels of prostaglandins, a hormone known to cause crampy abdominal pain. NSAIDs are medications that work by blocking the production of prostaglandins (5). NSAIDs are effective for treating dysmenorrhea, as demonstrated by a meta-analysis of 35 randomized controlled trials (5). However, a review of 51 different clinical trials found that 18% of women reported little to no relief from menstrual pain with NSAIDs (6). And NSAIDs carry a range of adverse effects, primarily affecting the gastrointestinal, renal, and cardiovascular systems (7). Given these limitations, non-pharmacological alternatives are increasingly sought.

A diverse range of non-pharmacological interventions exists, including dietary supplements, transcutaneous electrical nerve stimulation (TENS), acupuncture, and exercise (8–11). Among these options, thermal therapy stands out by enabling self-care for patients, offering a superior safety profile, and demonstrating high accessibility and public acceptance. The rationale for focusing on heat is 2-fold. First, it aligns with the prostaglandin-based pathophysiology of dysmenorrhea; applied heat increases pelvic blood flow, which may help to dissipate and reduce the concentration of prostaglandins, thereby relieving ischemia and muscle cramps (12). Second, it offers a unique combination of immediate, non-invasive analgesia and an exceptional safety profile, presenting a practical and accessible option for women seeking to avoid medication-related side effects (13). Therefore, we posit that thermal therapy represents a promising and strategic non-pharmacological approach worthy of in-depth study.

## 2 Methods

### 2.1 Literature search

An academic librarian systematically designed and executed comprehensive, database-specific search strategies for seven major biomedical databases: Cochrane Central Register of Controlled Trials (CENTRAL), PubMed, Web of Science, EMBASE, Chinese National Knowledge Infrastructure (CNKI), VIP Database for Chinese Technical Periodicals, and Wanfang Data. Our systematic search encompassed all available records from each database's inception through October 28, 2024 and updated to August 03, 2025, without imposing language or publication status limitations. We also searched the previous systematic reviews and screened the reference lists and the studies included (Supplementary Table 1).

### 2.2 Study selection

Pairs of reviewers (HZY, LXX, CZY, ZWY) independently screened titles, abstracts, and subsequently, the full texts of potentially eligible articles using standardized, pre-tested forms. The data extraction form was structured around the PICOS framework, covering participant characteristics (Population), detailed descriptions of the interventions and comparators (Intervention/Comparison), study design (Study), outcome measures (Outcome), along with risk of bias assessments and records of adverse effects (see Supplementary File 1).

Disagreements primarily concerned the applicability of the interventions or the certainty of outcome reporting in the full-text articles assessed. All disagreements were referred to the arbitrator (YDN). The arbitrator made the final decision by referring to the predetermined inclusion criteria outlined in the PICOS framework and based on the original article text.

We included trials that met the following criteria: (1) enrolled patients diagnosed with primary dysmenorrhea; (2) randomized participants to receive localized superficial heat therapy, defined as the application of any device or substance (e.g., electric heating pads, adhesive abdominal warmers, far-infrared belts, or moxibustion) aimed at transferring thermal energy continuously to the body, vs. a control (no treatment, placebo, or NSAIDs); (3) evaluated outcomes either in the immediate term (≤24 h) or analgesic effect or over the longer term (≥3 months) for repeated-use efficacy; and (4) reported measures of pain intensity or safety endpoints.

### 2.3 Data abstraction and risk of bias assessment

Four reviewers (YDN, LYY, HZY, CZY) extracted data from each eligible trial sequentially, ensuring they faced away from each other during the process. We gathered information on study characteristics, including author name, year of publication, study location, funding source, sample size, and length of follow-up, as well as intervention characteristics and all patient-important outcomes.

In cases where a study reported outcomes at multiple time points, we selected the most commonly reported follow-up period among the eligible trials. To account for within-person variability, we abstracted change scores from baseline; end scores were used only when change scores were not available. Additionally, when multiple instruments or questionnaires were employed to measure a common outcome (such as pain), we abstracted data solely for the most frequently used instrument across the eligible studies.

Three reviewers (HZY, LYY, CZY) independently assessed the risk of bias using a modified Cochrane Risk of Bias Tool 1.0 (14, 15). The tool assessed the following domains: random sequence generation; allocation concealment; blinding of study participants, healthcare providers, and outcome assessors; incomplete outcome data (≥20% missing data was considered high risk of bias); and other potential sources of bias. For each item, responses were scored as “definitely or probably yes” (low risk of bias) or “definitely or probably no” (high risk of bias). Disagreements between reviewers were resolved through discussion and, if necessary, by third-party adjudication (see Supplementary Table 2).

### 2.4 Data synthesis

For dichotomous outcomes, we calculated the relative risk (RR) and its corresponding 95% confidence interval (CI). For continuous outcomes, we calculated the weighted mean difference (WMD) and its corresponding 95%CI after we converted all the pain intensity data to the 10 cm visual analog scale (VAS) for pain (16). 1.5 cm was considered the minimal clinical important difference (MID) of pain intensity (17). We calculated the modeled risk difference (RD) value for comparisons to make the results easier to be understood.

We used a DerSimonian-Laird random effects model for all meta-analyses. Data were analyzed with STATA software version 17 (Stata Corp, College Station, TX, USA).

### 2.5 Certainty of evidence

We evaluated the certainty of evidence for all outcomes using the GRADE framework (Grading of Recommendations, Assessment, Development, and Evaluations) (18). Evidence from randomized controlled trials (RCTs) is initially rated as high certainty but was subject to downgrading by one or more levels (to moderate, low, or very low) following assessment across five domains: risk of bias, inconsistency, indirectness, imprecision, and publication bias—the latter evaluated through visual inspection of funnel plot asymmetry where ≥10 studies contributed to a meta-analysis. We defined it imprecise when 95% CIs of pain intensity contained either half MID (0.75 cm) or 0 cm, and 95% CIs of adverse events included no difference (RR = 1).

## 3 Results

### 3.1 Search results and study characteristics

**Figure 1:**
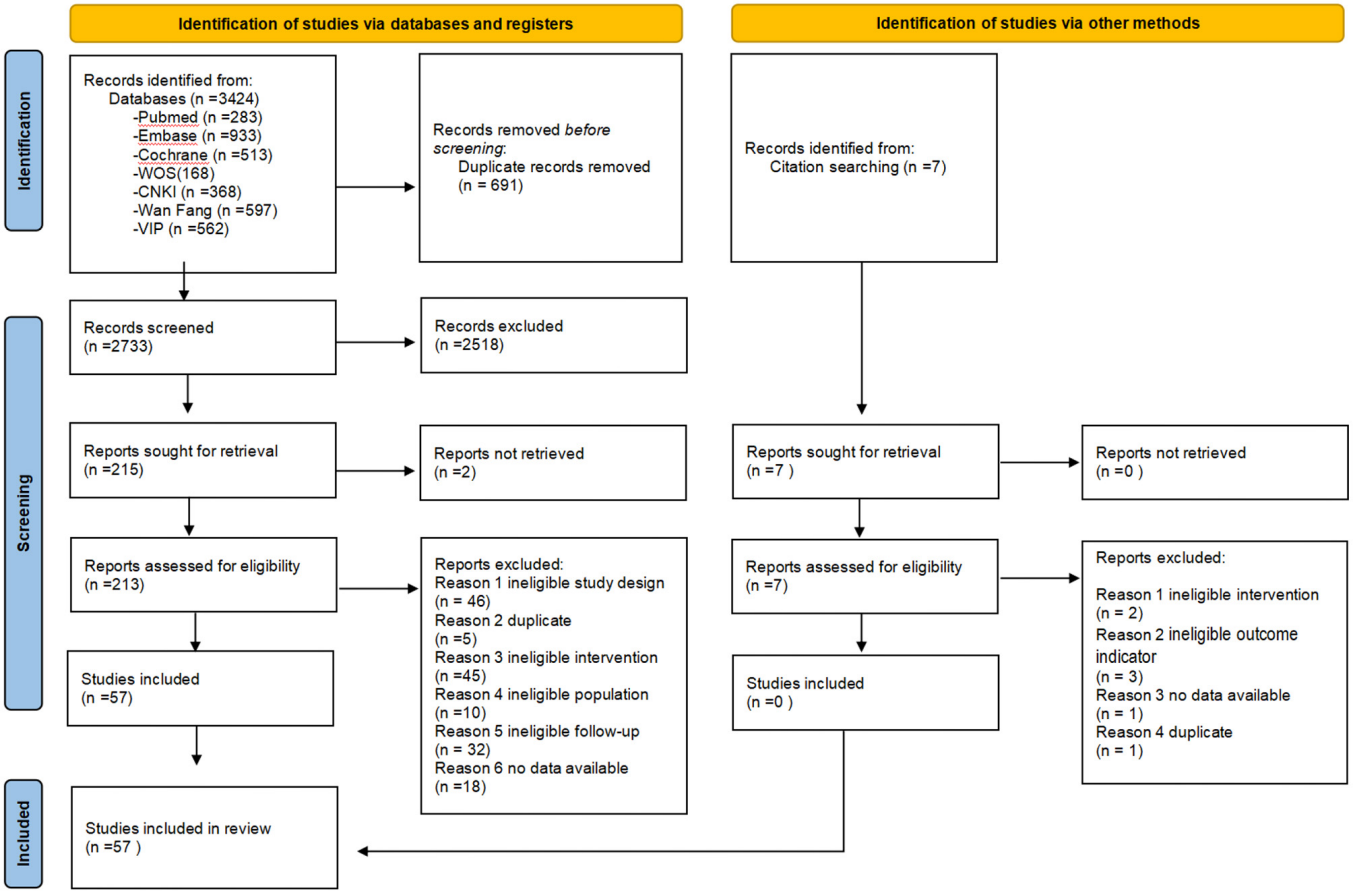
Search flowchart.

We screened 2,733 citations, identifying 57 eligible trials (19–75) involving 5,359 participants (search flow shown in Figure 1). The median of the mean ages reported across the 53 trials (19–25, 27–47, 49–57, 59–74) that provided age data was 22.3 years. Among the 44 trials (19–24, 27, 28, 30–34, 37–44, 47, 50–55, 57–64, 66, 68–74) reporting the duration of primary dysmenorrhea, the median of the mean durations was 44 months. Of the studies reporting location, 54 were conducted in Asia (19–28, 30–44, 46–74), two in Europe (29, 75), and one in South America (45). Twenty-eight trials (24–26, 29–34, 38, 40, 42, 45, 46, 48, 49, 52, 53, 55, 56, 59, 61, 64, 66–68, 73, 74) compared heat therapy with a control group [three (26, 29, 45) assessing short-term effects], and 24 trials (21–23, 27, 29, 35, 37, 39, 41, 44, 47, 50, 51, 54, 57, 58, 60, 62, 65, 69–72, 75) compared heat therapy with NSAIDs [two (29, 60) assessing short-term effects] (see Table 1).

**Table 1:** Baseline characteristics of included studies.

**Study**	**Intervention**	**Control**	**Funding**	**Country**	**Number of participants at baseline, *n***	**Length of follow-up, days**	**Mean duration of condition (SD), months**	**Mean age (SD), years**
Ma G 2002 (19)	Microwave therapy	Ibuprofen	NR	China	120	90	24 (4.23)	19 (2.73)
Zhang SM 2008 (20)	TDP & Moxi	Indometacin	NR	China	98	90	46.76(NR)	19.2 (NR)
Liu C 2011 (21)	Moxi	Ibuprofen	NR	China	80	90	68.2 (35.69)	21.22 (5.86)
Sun GY 2012 (22)	Moxi & Usual care	Ibuprofen & Usual care	NR	China	60	90	60 (NR)	23 (NR)
Lai J 2012 (23)	Super Lizer	Indometacin	NR	China	248	90	41.3 (17.02)	17.7 (2.25)
Li P 2012 (24)	Moxi	Blank	NR	China	50	90	74.6 (32.09)	21.9 (1.92)
Hou K 2013 (25)	Moxi & NSAID	NSAID	NR	China	78	90	NR	25.6(NR)
Li WJ 2013 (26)	Moxi	Blank	NR	China	76	20 min	NR	NR
Wen XR 2013 (27)	Moxi	Ibuprofen	NR	China	60	90	73.2 (36.6)	22.3 (2.53)
Zhu L 2013 (28)	Moxi & Acupuncture	Acupuncture	NR	China	60	90	72.7 (35.93)	22.3 (2.53)
Potur DC 2014 (29)	Hot post	NSAID or Blank	Government	Turkey	252	8h	NR	59.62 (1.18)
Jing XX 2015 (30)	Moxi & Ibuprofen	Ibuprofen	NR	China	100	90	27 (3.1)	22.2 (2.14)
Qian SH 2015 (31)	RDP & Point application theropy	Point application therapy	NR	China	52	90	41.3 (28.74)	21 (3.96)
Ou Y 2015 (32)	Moxi & TCM	TCM	NR	China	221	120	36 (NR)	21.2 (NR)
Zhu LH 2015 (33)	Moxi	Blank	Government	China	64	90	59.6 (20.25)	20.3 (1.6)
Li Y 2017 (34)	Moxi & Usual care	Usual care	Government	China	70	90	25.5 (12.58)	20.3 (1.05)
Yang MX 2017 (35)	Moxi	Ibuprofen	Government	China	152	90	NR	23 (2.92)
Hao MM 2017 (36)	Moxi	Painkiller	NR	China	80	90	NR	19.7 (NR)
Wang LY 2018 (37)	Moxi	Ibuprofen	NR	China	120	90	78.5 (39.76)	22.3 (2.63)
Chen ZH 2018 (38)	Moxi & Acupuncture	Acupuncture	NR	China	93	120	27.2 (14.23)	22.8 (3.1)
Li C 2018 (39)	Moxi	Ibuprofen	NR	China	72	90	54.7 (41.75)	23 (1.42)
Li XJ 2018 (40)	Moxi	Blank	Government	China	155	90	57 (5.39)	20 (0.5)
Song J 2018 (41)	Moxi	Ibuprofen	NR	China	60	90	60.1 (27.77)	23.6 (3.44)
Xian SW 2018 (42)	Moxi & Acupuncture	Acupuncture	Government	China	64	180	37.9 (32.13)	20.6 (1.42)
Yan LH 2018 (43)	Moxi & Ibuprofen	Ibuprofen	NR	China	106	90	5.4 (0.6)	24.7 (4.64)
Chen CX 2018 (44)	Moxi & Ibuprofen	Ibuprofen	Government	China	60	120	17.5 (7.05)	20.3 (1.62)
Machado AFP 2019 (45)	Thermal therapy & TENS	TENS	NR	Brazil	44	24 h	NR	22.6 (4.08)
Wang MJ 2019 (46)	Moxi & TCM	TCM	NR	China	60	90	NR	24.4 (NR)
Huang W 2019 (47)	Moxi	Ibuprofen	Government	China	100	90	36.8 (22.82)	20.4 (1.6)
Li L 2019 (48)	Moxi & Usual care	Usual care	NR	China	150	90	NR	NR
Liao BD 2019 (49)	Moxi & Needle warming Moxi	Needle warming Moxi	NR	China	120	120	NR	24 (3)
Jiang M 2020 (50)	Moxi	Ibuprofen	Government	China	60	90	30.4 (4.42)	22.2 (4.03)
Liu Q 2020 (51)	Moxi	Ibuprofen	Government	China	100	90	48.2 (14.42)	26.3 (4.98)
Liu LY 2020 (52)	Moxi	Blank	Government	China	144	90	54 (6.8)	20 (0.5)
Sun L 2020 (53)	Moxi & TCM	TCM	NR	China	72	90	68 (27.45)	26 (3.75)
Wei MP 2020 (54)	Moxi	Ibuprofen	NR	China	102	90	23.4 (7.04)	20.1 (2.34)
Zhou WY 2020 (55)	Moxi & TCM	TCM	NR	China	146	90	38.6 (13.01)	26.7 (1.87)
Song H 2021 (56)	Moxi & Acupuncture and cupping	Acupuncture and cupping	NR	China	127	90	NR	22.2 (2.88)
Wei XH 2021 (57)	Moxi	Ibuprofen	NR	China	80	90	32.9 (13.53)	20.4 (1.83)
Pan WB 2022 (58)	Moxi	Ibuprofen	Government	China	99	90	39.5 (18.83)	NR
Wang GQ 2022 (59)	Moxi & TCM	TCM	NR	China	90	90	23.4 (7.33)	21 (2.62)
Liang H 2022 (60)	Electromagnetic wave	Ibuprofen	NR	China	40	30 min	34 (4.73)	19.2 (1.02)
Yang JQ 2022 (61)	Moxi & TCM	TCM	NR	China	62	90	86.7 (35.72)	27 (3.04)
Yang YF 2022 (62)	Moxi	Ibuprofen	NR	China	60	90	47.6 (5.23)	35 (5.19)
Zhan L 2022 (63)	Moxi	Ibuprofen	NR	China	104	90	19.1 (14.58)	23.4 (2.77)
Shen JW 2023 (64)	Moxi patch & TCM	TCM	Government	China	60	90	49.2 (22.86)	29.4 (4.14)
Lin SF 2023 (65)	TDP & Moxi	Indometacin	NR	China	50	90	NR	18.1 (3.5)
Ma TT 2023 (66)	Moxi patch & TCM	TCM	NR	China	90	180	58.8 (39.49)	27.6 (5.32)
Wu JJ 2023 (67)	Moxi & Usual care	Usual care	Government	China	76	90	NR	22 (2.48)
Lin WM 2023 (68)	Moxi	Ibuprofen	Government	China	120	90	80.8 (38.49)	24.9 (6.05)
Xing BB 2023 (69)	Moxi	Ibuprofen	NR	China	66	90	81 (32.28)	25.2 (2.56)
Yu SY 2024 (70)	Moxi patch & TCM	TCM	NR	China	88	90	12.5 (3.4)	26.4 (3.26)
Yang SR 2024 (71)	Moxi	Ibuprofen	Government	China	120	90	84 (NR)	26.3 (NR)
Xu YY 2024 (72)	Moxi	Ibuprofen	NR	China	68	90	142 (77.94)	29.4 (6.49)
Chen Y 2024 (73)	Moxi & Acupuncture	Acupuncture	NR	China	64	90	10.1 (8.33)	25.1 (4.02)
Qiu J 2025 (74)	Moxi & catgut embedding	Catgut embedding	Government	China	90	90	8.4 (1.74)	23.4 (2.6)
Ceylan D 2025 (75)	Thermal therapy	Dexketoprofen trometamol	Government	Turkey	56	90	NR	NR

Moxi, moxibustion; TCM, traditional Chinese medicine; TDP, Thermal Diffusion Therapy.

### 3.2 Risk of bias

The risk of bias assessment for the 57 included trials is summarized in Supplementary Table 2. Random sequence generation was adequately reported in 36 trials (63%) (21, 27–29, 31–35, 39–42, 44, 45, 47, 49–53, 55–58, 60, 62, 64–69, 71, 72, 74), suggesting a low risk of selection bias for this domain in these studies. However, allocation concealment was implemented in only 17 trials (30%) (21, 27–29, 31–35, 39–42, 45, 52, 55, 74), potentially compromising 1.5 cm was considered the minimal clinica integrity. Only 3 trials (5%) (45, 52, 74) blinded participants, and 3 (5%) (28, 40, 45) blinded healthcare providers. This high risk of performance bias means that the expectation of receiving a therapeutic intervention (heat) could have influenced participants' reporting of pain relief. Similarly, blinding of outcome assessors and data analysts was reported in only 6 trials (11%) (28, 33, 35, 40, 41, 52), constituting a significant source of detection bias for the subjective outcome of self-reported pain. Importantly, no trials had ≥20% missing data, which minimizes bias from incomplete outcomes and strengthens the robustness of the pooled analysis.

### 3.3 Heat therapy vs. blank control

#### 3.3.1 Pain analgesia over 3 months

**Figure 2:**
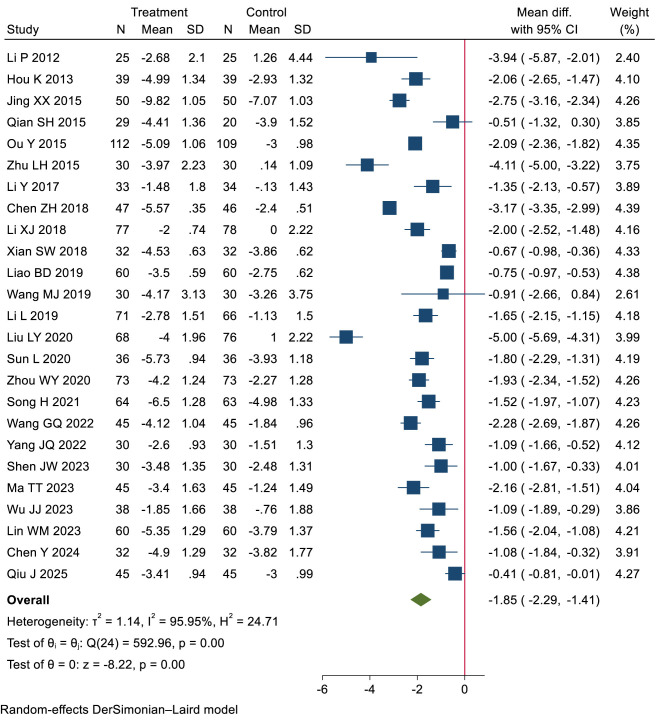
Long-term pain relief: heat therapy group vs. blank group.

Low-certainty evidence (25 RCTs, 2,393 patients) (24, 25, 30–34, 38, 40, 42, 46, 48, 49, 52, 53, 55, 56, 59, 61, 64, 66–68, 73, 74) showed that compared with blank intervention, patients with dysmenorrhea who received heat treatment may have experienced more pain relief (WMD −1.85 cm, 95% CI −2.29 to −1.41 cm; the modeled RD 21%, 95% CI 19% to 22%) (see Tables 2, 3; Figure 2).

**Table 2:** Summary of key findings: heat therapy vs. control/NSAIDs for primary dysmenorrhea.

**Comparison**	**Outcome**	**Time point**	**Certainty of evidence**	**Result (heat vs. comparator)**
Heat vs. blank control	Pain relief (VAS, cm)	≥3 months	Low	Superior to control (WMD −1.85 cm, 95% CI: −2.29 to −1.41)
	Pain relief (VAS, cm)	≤24 h	Low	Superior to control (WMD −3.52 cm, 95% CI: −5.01 to −2.02)
	Adverse effects	Various	Low	Little to no difference (RR 1.34, 95% CI: 0.44 to 4.16)
Heat vs. NSAIDs	Pain relief (VAS, cm)	≥3 months 24 h	Low	Similar efficacy (WMD −1.10 cm, 95% CI: −1.51 to −0.70)
	Pain relief (VAS, cm)	≤24 h	Low	Similar efficacy (WMD −1.5 cm, 95% CI: −2.86 to −0.15)
	Adverse effects	Various	Moderate	Safer than NSAIDs (RR 0.3, 95% CI: 0.15 to 0.59)

**Table 3:** Grade evidence profile of Heat therapy vs. the blank control on primary dysmenorrhea.

**No. of trials (No. of patients)**	**Follow-up, weeks**	**Risk of bias**	**Inconsistency**	**Indirectness**	**Imprecision**	**Publication bias**	**Treatment association (95% CI)**		**Overall quality of evidence**
**Pain: 0–10 cm VAS for pain (3 months effect); lower is better; MID** = **1.5 cm**									
25 (2,393)		Serious^a^	Not serious^b^, I^2^=95.95%	Not serious	Not serious	Serious^c^	Achieved at or above MID		
							Heat 98%	Control 77%	Low
							Modeled RD 21% (19%, 22%)		
							WMD −1.85 (−2.29, −1.41)		
**Pain: 0–10 cm VAS for pain (24 h effect); lower is better; MID** = **1.5 cm**									
3 (248)		Serious^a^	Not serious^b^, *I*^2^ = 89.10%	Not serious	Serious^d^	NA	Achieved at or above MID		Low
							Heat 96%	Control 51%	
							Modeled RD 45% (33%, 48%)		
							WMD −3.52 (−5.01, −2.02)		
**Adverse effects**									
7 (784)		Serious^a^	Not serious, *I*^2^ = 43.4%	Not serious	Serious^e^	NA	RR 1.35 (0.44, 4.16)		Low

NA, not available. ^a^High risk of bias in blinding and randomization ^b^Heterogeneity of more than 50%, but all research effects pointed in the same direction and there were only variations in effect sizes ^c^Visual inspection of the funnel plot indicated asymmetry, suggesting a potential risk of bias (see Supplementary Figure 1). ^d^Small sample size. ^e^95%CI cross the null line.

#### 3.3.2 Pain analgesia within 24 h

**Figure 3:**
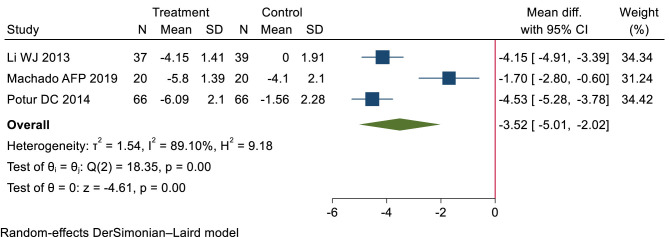
Short-term pain relief: heat therapy group vs. blank group.

Low-certainty evidence (3 RCTs, 248 patients) (26, 29, 45) suggested that compared with blank intervention, patients with dysmenorrhea who received heat treatment experienced more pain relief (WMD −3.52 cm, 95% CI −5.01 to −2.02 cm; the modeled RD 45%, 95% Cl 33% to 48%) (see Tables 2, 3; Figure 3).

#### 3.3.3 Adverse effects

**Figure 4:**
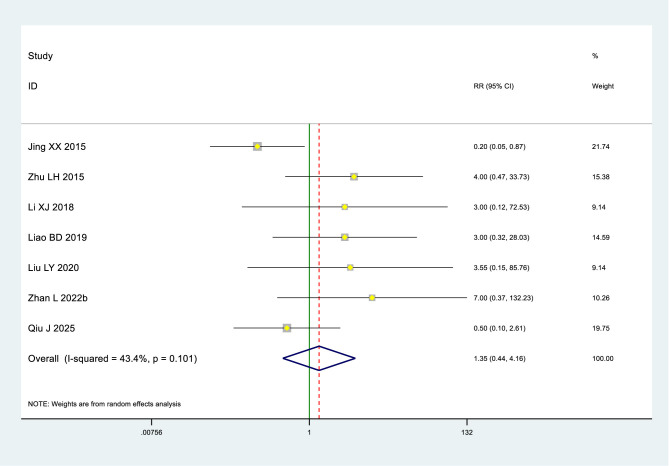
Adverse effects in the heat therapy and bank groups.

Low-certainty evidence (7 RCTs, 784 patients) (30, 33, 40, 49, 52, 63, 74) indicated little to no difference in adverse effects between heat therapy and blank intervention for primary dysmenorrhea (RR 1.34, 95% Cl 0.44–4.16) (see Tables 2, 3; Figure 4).

### 3.4 Heat therapy vs. NSAIDs

#### 3.4.1 Pain analgesia over 3 months

**Figure 5:**
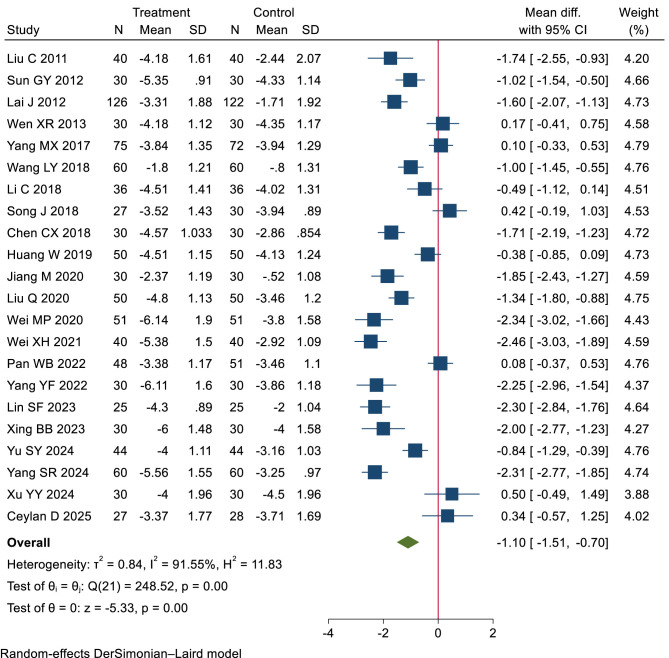
Long-term pain relief: heat therapy group vs. NSAIDs group.

Low-certainty evidence (22 RCTs, 1,938 patients) (21–23, 27, 35, 37, 39, 41, 44, 47, 50, 51, 54, 57, 58, 62, 65, 69–72, 75) suggested that heat therapy and NSAIDs may be comparable in relieving pain, with WMD −1.10 cm (95% CI −1.51 to −0.70 cm), modeled RD 4% (95% CI 3% to 4%) (see Tables 2, 4; Figure 5).

**Table 4:** Grade evidence profile of Heat therapy vs. medication on primary dysmenorrhea.

**No. of trials (No. of patients)**	**Follow-up, weeks**	**Risk of bias**	**Inconsistency**	**Indirectness**	**Imprecision**	**Publication bias**	**Treatment association (95% CI)**		**Overall quality of evidence**
**Pain: 0–10 cm VAS for pain (long-lasting effect); lower is better; MID** = **1.5 cm**									
22 (1,938)		Serious^a^	Not serious^b^, *I*^2^ = 91.55%	Not serious	Not serious	Serious^c^	Achieved at or above MID	
							Heat 100%	Control 96%	Low
							Modeled RD 4% (3%, 4%)		
							WMD −1.10 (−1.51, −0.70)		
**Pain: 0–10 cm VAS for pain (short-lasting effect); lower is better; MID** = **1.5 cm**									
2 (167)		Serious^d^	Not serious^e^, *I*^2^ = 80.77%	Not serious	Serious^f^	NA	Achieved at or above MI		Low
							Heat 93%	Control 77%	
							Modeled RD 16% (2%, 21%)		
							WMD −1.50 (−2.86, −0.15)		
**Adverse effects**									
8 (728)		Serious^a^	Not serious, *I*^2^ = 0%	Not serious	Not serious	NA	RR 0.30 (0.15, 0.59)		Moderate

NA, not available. ^a^High risk of bias in blinding and randomization. ^b^Heterogeneity of more than 50%, but all research effects pointed in the same direction and there were only variations in effect sizes. ^c^Visual inspection of the funnel plot indicated asymmetry, suggesting a potential risk of bias (see Supplementary Figure 2). ^d^High risk of bias in blinding. ^e^We downgraded for Imprecision but not for Inconsistency, as we judged the consistent results here would not substantially impact the overall findings. ^f^Small sample size and 95% CI cross the half of the MID value.

#### 3.4.2 Pain analgesia within 24 h

**Figure 6:**
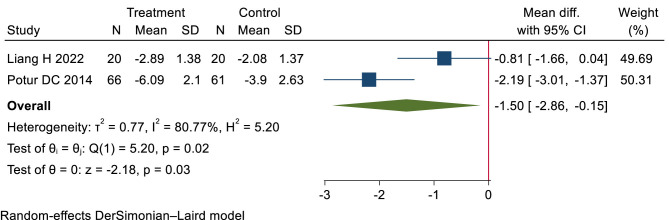
Short-term pain relief: heat therapy group vs. NSAIDs group.

Low-certainty evidence (2 RCTs, 167 patients) (29, 60) suggested that heat therapy and NSAIDs may show similar efficacy in pain relief, with WMD −1.5 cm (95% CI −2.86 to −0.15 cm), modeled RD 16% (95% CI 2% to 21%) (see Tables 2, 4; Figure 6).

#### 3.4.3 Adverse effects

**Figure 7:**
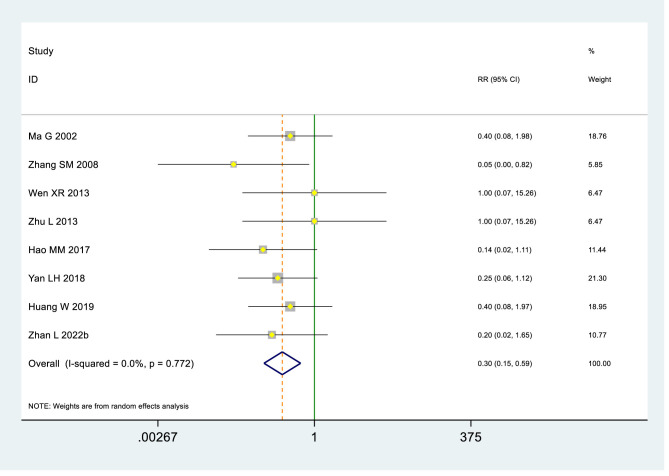
Adverse effects in the Heat therapy and NSAIDs groups.

Moderate-certainty evidence (8 RCTs, 728 patients) (19, 20, 27, 28, 36, 43, 47, 63) indicated that heat therapy probably reduced the risk of adverse effects compared with NSAIDs in primary dysmenorrhea (RR 0.3, 95% Cl 0.15 to 0.59) (see Tables 2, 4; Figure 7).

## 4 Discussion

### 4.1 Overall findings

Compared to no treatment, heat therapy reduces pain in primary dysmenorrhea with comparable safety. When compared to NSAIDs, heat therapy demonstrates minimal difference in pain intensity but is probably associated with fewer adverse events. These treatment outcomes remain consistent across both short-term (24-h) and long-term (3-month) assessments.

### 4.2 Relation to other studies

We have identified two systematic reviews in the literature addressing heat therapy for primary dysmenorrhea (76, 77); however, we excluded 6 RCTs for specific reasons. The first meta-analysis (76) included three RCTs on thermotherapy. One trial was excluded due to a lack of baseline data (78). The other two (79, 80), with treatment durations of 1 and 2 months, were also excluded. The second meta-analysis (77) included six RCTs (29, 78, 80–83), only one of which was included in our analysis (29). The other five trials were excluded due to the absence of extractable outcome measures (78, 80, 81), unavailable resources (82), or non-compliant interventions (83). A detailed breakdown is provided in Supplementary Table 3.

Earlier systematic reviews offered valuable preliminary insights by suggesting heat therapy might be effective and potentially comparable to analgesic medication. However, their conclusions were notably constrained: Jo and Lee's analysis, while indicating superiority over placebo, was limited to only 6 RCTs (77); Igwea et al. identified merely 3 heat therapy trials, were unable to perform a direct comparative meta-analysis, and ultimately highlighted the need for more robust evidence (76).

Our study comprehensively addresses these limitations through key advancements: a markedly expanded evidence base (57 RCTs) enabling more precise and generalizable treatment estimates; broader intervention diversity encompassing microwave therapy, electromagnetic wave therapy, moxibustion, and hot packs beyond previous narrow focus; and demonstration of consistent therapeutic benefits across both immediate (24-h) and sustained (3-month) timeframes—a previously unexamined dimension. Methodologically, the systematic application of the GRADE framework provides rigorous evidence certainty assessment, thereby substantiating prior hypotheses and establishing a more reliable foundation for positioning heat therapy as a viable non-pharmacological treatment for primary dysmenorrhea.

### 4.3 Strengths and limitations

This review has several strengths. We predefined the MIDs to visually demonstrate between-group differences and evaluate the clinical significance of the findings. The calculation of MID-derived risk differences (RDs) further enhanced the interpretation of the clinical feasibility of treatment effects. Moreover, our study overcomes the limitations of previous analyses by comprehensively incorporating diverse thermotherapy modalities and leveraging a robust sample size, thereby yielding more precise and generalizable estimates.

Nevertheless, several limitations warrant consideration. The methodological quality of many included trials was compromised by inadequate randomization and concealment of allocation. Furthermore, significant heterogeneity was observed in some analyses, likely stemming from clinical diversity in patient populations and variations in heat therapy protocols. Finally, the predominance of studies conducted in Asian populations may limit the generalizability of our findings to other regions.

### 4.4 Implications

Our findings provide evidence for informing a stepped-care approach to managing primary dysmenorrhea. During acute episodes, local heat therapy using modalities such as hot water bottles or self-heating patches can provide immediate pain relief comparable to NSAIDs, with a superior safety profile. This offers an ideal first-line option for patients who cannot or prefer not to use medication. During the intermenstrual period, regular application of heat therapies like moxibustion or infrared therapy can serve as an effective preventive measure. Long-term adherence may reduce the frequency and intensity of pain episodes and decrease reliance on analgesic medications. For patients with severe pain, a “heat therapy-first, medication-as-supplement” combination strategy could be considered—employing heat therapy both preventively and during acute phases, reserving short-term NSAID use only for peak pain levels to optimize both efficacy and safety.

From a research perspective, the application of MIDs in our meta-analysis offers a concrete method for evaluating the clinical significance of future findings. However, the promising results are constrained by the low certainty of evidence and prevalent risk of bias in existing studies.

Future research should therefore prioritize high-quality, adequately powered RCTs that are specifically designed to overcome these limitations. We recommend that future trials: (1) calculate sample sizes based on the established MIDs for pain scales to ensure sufficient statistical power; (2) predefine and consistently apply a standardized heat intervention protocol (specifying temperature, application site, duration, and treatment frequency) to reduce heterogeneity; (3) adhere to the CONSORT reporting guidelines, providing clear descriptions of randomization, allocation concealment, and blinding methods; and (4) systematically record and report all adverse events to better establish the long-term safety profile of repeated heat application. Such rigorously generated evidence is crucial to confirm these findings and establish clear, evidence-based clinical guidelines.

## 5 Conclusion

Compared to no treatment, heat therapy is likely to reduce pain intensity both during prophylaxis and acute episodes. When compared to NSAIDs, heat therapy may achieve comparable analgesic efficacy with a superior safety profile.

## Data Availability

The original contributions presented in the study are included in the article/Supplementary material, further inquiries can be directed to the corresponding author.
