# Supplementary material for: Heat therapy for primary dysmenorrhea: a systematic review and meta-analysis
Source: Front Med (Lausanne). 2026 Jan 23;12:1730505. doi: 10.3389/fmed.2025.1730505 (PMC12876241; doi:10.3389/fmed.2025.1730505)
Supplement: Supplementary file 1 [file Data_Sheet_1.zip › Add supplementary charts/Supplement Table 1.docx]

**Supplement Table 1. Literature Search Strategies in Databases**

**Supplement Table 1.1 Pubmed search <inception to October 28, 2024>**

| **Search Strategy and Search Results** |
| --- |
| **inception to October 28, 2024** |
| #1  dysmenorrhea[MeSH Terms] OR Dysmenorrheas OR period pain[MeSH Terms] OR menstrual pain[MeSH Terms] OR menstrual cramps[MeSH Terms] OR painful menstruation[MeSH Terms] OR painful periods[MeSH Terms] OR Menstruation, Painful[MeSH Terms] OR Pain, Menstrual[MeSH Terms] OR cramp[MeSH Terms] (8135)  #2  dysmenorrhea[Title/Abstract] OR period pain[Title/Abstract] OR menstrual pain[Title/Abstract] OR menstrual cramps[Title/Abstract] OR painful menstruation[Title/Abstract] OR painful periods[Title/Abstract] OR Pain, Menstrual[Title/Abstract] OR cramp[Title/Abstract] OR Menstruations, Painful[Title/Abstract] OR Painful Menstruations[Title/Abstract] OR Menstrual Pain[Title/Abstract] OR menstrual period pain[Title/Abstract] OR painful catamenia[Title/Abstract] (7508)  #3 #1 OR #2 (9858)  #4 heat therapy[MeSH Terms] OR Infrared Ray[MeSH Terms] OR Moxibustion[MeSH Terms] OR Hot Temperatures[MeSH Terms](137987)  #5 Thermotherapy OR Fever Therapy OR Induced Hyperthermia OR Therapeutic Hyperthermia OR Local Hyperthermia OR Heat Wave OR Moxabustion OR Moxibustion OR Hyperthermia OR Temperature OR Hot OR Heat OR Infrared Ray OR ammotherapy OR hyperthermia, induced OR infra red therapy OR infrared radiation OR infrared (1468095)  #6 #4 OR #5 (1468095)  #7 #3 AND #6 (527)  #8  Placebos[MeSH Terms] or 'Randomized Controlled Trial' or 'Clinical Study' or randomized controlled trial OR controlled clinical trial OR placebo OR random* OR trial OR groups OR Clinical randomised controlled trial OR randomised control OR randomized OR RCT or clinical trial (6811356)  #9 #7 AND #8 (266) |
| **October 28, 2024 to August 3, 2025** |
|  |

#1 dysmenorrhea[MeSH Terms] OR Dysmenorrheas OR period pain[MeSH Terms] OR menstrual pain[MeSH Terms] OR menstrual cramps[MeSH Terms] OR painful menstruation[MeSH Terms] OR painful periods[MeSH Terms] OR Menstruation, Painful[MeSH Terms] OR Pain, Menstrual[MeSH Terms] OR cramp[MeSH Terms] (8579)

#2 dysmenorrhea[Title/Abstract] OR period pain[Title/Abstract] OR menstrual pain[Title/Abstract] OR menstrual cramps[Title/Abstract] OR painful menstruation[Title/Abstract] OR painful periods[Title/Abstract] OR Pain, Menstrual[Title/Abstract] OR cramp[Title/Abstract] OR Menstruations, Painful[Title/Abstract] OR Painful Menstruations[Title/Abstract] OR Menstrual Pain[Title/Abstract] OR menstrual period pain[Title/Abstract] OR painful catamenia[Title/Abstract] (8016)

#3 #1 OR #2 (10397)

#4 heat therapy[MeSH Terms] OR Infrared Ray[MeSH Terms] OR Moxibustion[MeSH Terms] OR Hot Temperatures[MeSH Terms](142109)

#5 Thermotherapy OR Fever Therapy OR Induced Hyperthermia OR Therapeutic Hyperthermia OR Local Hyperthermia OR Heat Wave OR Moxabustion OR Moxibustion OR Hyperthermia OR Temperature OR Hot OR Heat OR Infrared Ray OR ammotherapy OR hyperthermia, induced OR infra red therapy OR infrared radiation OR infrared (1535040)

#6 #4 OR #5 (1535040)

#7 #3 AND #6 (557)

#8 Placebos[MeSH Terms] or 'Randomized Controlled Trial' or 'Clinical Study' or randomized controlled trial OR controlled clinical trial OR placebo OR random* OR trial OR groups OR Clinical randomised controlled trial OR randomised control OR randomized OR RCT or clinical trial(7147720)

#9 #7 AND #8 (283)

#10 "2024/10/29"[Date - Publication] : "2025/08/03"[Date - Publication]] (17)

**Supplement Table 1.2 Embase search <inception to March 13, 2025>**

| **Search Strategy and Search Results** |
| --- |
| **inception to October 28, 2024** |
| #1 'randomized controlled trial'/exp OR 'controlled trial, randomized' OR 'randomised controlled study' OR 'randomised controlled trial' OR 'randomized controlled study' OR 'trial, randomized controlled' OR 'randomized controlled trial' OR 'clinical trial'/exp OR 'clinical drug trial' OR 'major clinical trial' OR 'trial, clinical' OR 'clinical trial' OR 'placebo'/exp OR 'placebo gel' OR 'placebos' OR 'placebo' OR 'clinical study'/exp OR 'clinical data' OR 'clinical studies as topic' OR 'medical trial' OR 'clinical study'（13605912）  #2  'dysmenorrhea '/exp （17231）  #3 'dysmenorrhea' OR 'period pain*' OR 'menstrual pain*' OR 'menstrual cramps' OR 'painful menstruation*' OR 'painful periods' OR 'catamenial pain*' OR 'dys-menorrhea' OR 'catamenial pain*' OR 'painful catamenia'（19953）  #4 #2OR#3：（19953）  #5  'thermotherapy'/exp OR 'infrared radiation '/exp OR 'moxibustion'/exp（89881）  #6 'thermotherapy' OR 'ammotherapy' OR 'hyperthermia' OR 'dry heat therapy' OR 'heat therapy' OR 'hyperthermic therapy' OR 'hyperthermic treatment' OR 'induced hyperthermia' OR 'infrared therapy' OR 'thermal therapy' OR 'thermotherapy' OR 'infrared radiation' OR 'infrared' OR 'infrared emission' OR 'infrared light emission' OR 'infrared ray' OR 'infrared spectra' OR 'moxibustion' OR 'hot' OR 'temperature' OR 'heat' （1860436）  #7 #5 OR #6:（1869796）  #8 #1and #4 and #7 （829） |
| **October 28, 2024 to August 3, 2025** |
| #1 'randomized controlled trial'/exp OR 'controlled trial, randomized' OR 'randomised controlled study' OR 'randomised controlled trial' OR 'randomized controlled study' OR 'trial, randomized controlled' OR 'randomized controlled trial' OR 'clinical trial'/exp OR 'clinical drug trial' OR 'major clinical trial' OR 'trial, clinical' OR 'clinical trial' OR 'placebo'/exp OR 'placebo gel' OR 'placebos' OR 'placebo' OR 'clinical study'/exp OR 'clinical data' OR 'clinical studies as topic' OR 'medical trial' OR 'clinical study'（14951143）  #2 'dysmenorrhea '/exp （19024）  #3 'dysmenorrhea' OR 'period pain*' OR 'menstrual pain*' OR 'menstrual cramps' OR 'painful menstruation*' OR 'painful periods' OR 'catamenial pain*' OR 'dys-menorrhea' OR 'catamenial pain*' OR 'painful catamenia'（21900）  #4 #2OR#3：（21900）  #5 'thermotherapy'/exp OR 'infrared radiation '/exp OR 'moxibustion'/exp（99917）  #6 'thermotherapy' OR 'ammotherapy' OR 'hyperthermia' OR 'dry heat therapy' OR 'heat therapy' OR 'hyperthermic therapy' OR 'hyperthermic treatment' OR 'induced hyperthermia' OR 'infrared therapy' OR 'thermal therapy' OR 'thermotherapy' OR 'infrared radiation' OR 'infrared' OR 'infrared emission' OR 'infrared light emission' OR 'infrared ray' OR 'infrared spectra' OR 'moxibustion' OR 'hot' OR 'temperature' OR 'heat' （1987032）  #7 #5 OR #6:（1997787）  #8 #1and #4 and #7 （933）  #9 limit #8 to [29-10-2024]/sd NOT [04-08-2025]/sd AND [2024-2025]/py (104) |

**+**

**Supplement Table 1.3 Web of Science search <inception to January 8, 2025>**

| **Search Strategy and Search Results** |
| --- |
| **inception to October 28, 2024** |
| #1 TS=(dysmenorrhea*) OR TS=("period pain*") OR TS=(menstrual cramps) OR TS=(painful menstruation*) OR TS=("painful periods") OR TS=(catamenial pain*) OR TS=(menstrual period pain) OR TS=(menstrual pain*) OR TS=(catamenial pain*) OR TS=(painful catamenia*) (5502)  #2 TS=(Thermotherapy) OR TS=("Fever Therapy") OR TS=("Heat Wave*") OR TS=(Moxibustion) OR TS=(Hyperthermia) OR TS=("Temperature") OR TS=("Hot") OR TS=("Heat") OR TS=("Infrared Ray") OR TS=(ammotherapy) OR TS=("infra red therapy") OR TS=("infrared radiation") OR TS=(infrared) (3091197)  #3  TS=(Placebos) OR TS=("Randomized Controlled Trial") OR TS=("Clinical Study") OR TS=(randomized controlled trial) OR TS=(controlled clinical trial) OR TS=(placebo) OR TS=(random*) OR TS=(trial) OR TS=(groups) OR TS=(Clinical randomised controlled trial) OR TS=(corpulent) OR TS=(randomised control) OR TS=(randomized) OR TS=(RCT) OR TS=(clinical trial) (5021396)  #4 #1 and #2 and #3 (161) |
| **October 28, 2024 to August 3, 2025** |
| #1 TS=(dysmenorrhea*) OR TS=("period pain*") OR TS=(menstrual cramps) OR TS=(painful menstruation*) OR TS=("painful periods") OR TS=(catamenial pain*) OR TS=(menstrual period pain) OR TS=(menstrual pain*) OR TS=(catamenial pain*) OR TS=(painful catamenia*) (6184)  #2 TS=(Thermotherapy) OR TS=("Fever Therapy") OR TS=("Heat Wave*") OR TS=(Moxibustion) OR TS=(Hyperthermia) OR TS=("Temperature") OR TS=("Hot") OR TS=("Heat") OR TS=("Infrared Ray") OR TS=(ammotherapy) OR TS=("infra red therapy") OR TS=("infrared radiation") OR TS=(infrared) (3341229)  #3 TS=(Placebos) OR TS=("Randomized Controlled Trial") OR TS=("Clinical Study") OR TS=(randomized controlled trial) OR TS=(controlled clinical trial) OR TS=(placebo) OR TS=(random*) OR TS=(trial) OR TS=(groups) OR TS=(Clinical randomised controlled trial) OR TS=(corpulent) OR TS=(randomised control) OR TS=(randomized) OR TS=(RCT) OR TS=(clinical trial) (5442274)  #4 #1 and #2 and #3 (168)  #5 #4 Timespan: 2024-10-29 to 2025-08-03 (Index Date) (11) |

**Supplement Table 1.4 Cochran <inception to January 8, 2025>**

| **Search Strategy and Search Results** |
| --- |
| **inception to October 28, 2024** |
| #1  dysmenorrhea[MeSH Terms](952)  #2  （title/ab/keywords）dysmenorrhea* OR "period pain" OR menstrual pain* OR menstrual cramps OR painful menstruation OR "painful periods" OR "catamenial pain" OR dys-menorrh*ea OR "menstrual period pain" OR "catamenial pain" OR "painful catamenia"(5057)  #3  #1 OR #2(5057)  #4  Thermotherapy[MeSH Terms](2501)  #5 （title/ab/keywords）Thermotherapy OR "Fever Therapy" OR "Induced Hyperthermia" OR "Therapeutic Hyperthermia" OR "Local Hyperthermia" OR "Heat Wave" OR Moxabustion OR Moxibustion OR Hyperthermia OR Temperature OR Hot OR Heat OR "Infrared Ray" OR ammotherapy OR "hyperthermia, induced" OR "infra red therapy" OR "infrared radiation" OR infrared（53508）  #6 #4 OR #5（54984）  #7  Placebos or "Randomized Controlled Trial" or "Clinical Study" or randomized controlled trial OR controlled clinical trial OR placebo OR random* OR trial OR groups OR Clinical randomised controlled trial OR randomised control OR randomized OR RCT or clinical trial（1748539）  #8 #3 AND #6 AND #7 (435) |
| **October 28, 2024 to August 3, 2025** |
| #1 dysmenorrhea[MeSH Terms](988)  #2 （title/ab/keywords）dysmenorrhea* OR "period pain" OR menstrual pain* OR menstrual cramps OR painful menstruation OR "painful periods" OR "catamenial pain" OR dys-menorrh*ea OR "menstrual period pain" OR "catamenial pain" OR "painful catamenia"(5699)  #3 #1 OR #2(5699)  #4 Thermotherapy[MeSH Terms](2515)  #5 （title/ab/keywords）Thermotherapy OR "Fever Therapy" OR "Induced Hyperthermia" OR "Therapeutic Hyperthermia" OR "Local Hyperthermia" OR "Heat Wave" OR Moxabustion OR Moxibustion OR Hyperthermia OR Temperature OR Hot OR Heat OR "Infrared Ray" OR ammotherapy OR "hyperthermia, induced" OR "infra red therapy" OR "infrared radiation" OR infrared（57588）  #6 #4 OR #5（59094）  #7 Placebos or "Randomized Controlled Trial" or "Clinical Study" or randomized controlled trial OR controlled clinical trial OR placebo OR random* OR trial OR groups OR Clinical randomised controlled trial OR randomised control OR randomized OR RCT or clinical trial（1961059）  #8 #3 AND #6 AND #7 (513)  #9 #8 with Cochrane Library publication date from Nov 2024 to Aug 2025 (36) |

**Supplement Table 1.5 CNKI/Wan Fang/VIP/Sinomed search <inception to August 3, 2025>**

| **Search Strategy and Search Results** |
| --- |
| #1 Dysmenorrhea OR Menstrual period abdominal pain OR Menstrual pain OR cramp OR premenstrual abdominal pain OR Post-abdominal pain  #2 Thermal stimulus OR Infrared ray OR Infrared radiation OR Frequency spectrum OR Moxibustion OR TDP OR Special electro-magnetic therapeutic apparatus OR External radiot herapy OR Thermotherapy OR HeTrmal therapy  #3 Random OR Contrast OR Randomized controlled OR Blank OR Clinical test OR Clinic trial  #4 #1 AND #2 AND #3 |
