# Supplementary material for: Heat therapy for primary dysmenorrhea: a systematic review and meta-analysis
Source: Front Med (Lausanne). 2026 Jan 23;12:1730505. doi: 10.3389/fmed.2025.1730505 (PMC12876241; doi:10.3389/fmed.2025.1730505)
Supplement: Supplementary file 1 [file Data_Sheet_1.zip › Add supplementary charts/Supplement Table 3.docx]

| **Reference ID** | **RCT** | **Reasons to exclude** |
| --- | --- | --- |
| 1 | Ke 2012 | no data available |
| 2 | Akin 2001 | ineligible outcome indicator |
| 3 | Akin 2004 | ineligible outcome indicator |
| 4 | Navvabi 2012 | ineligible outcome indicator |
| 5 | Lee 2011 | ineligible intervention |
| 6 | Kim JI 2013 | ineligible intervention |

**Supplement Table 3. The reasons to exclude included studies of previous systematic reviews of ACE for PCOS**
