# Supplementary figures and images for: Heat therapy for primary dysmenorrhea: a systematic review and meta-analysis
Source: Front Med (Lausanne). 2026 Jan 23;12:1730505. doi: 10.3389/fmed.2025.1730505 (PMC12876241; doi:10.3389/fmed.2025.1730505)

Funnel plot with pseudo 95% confidence limits

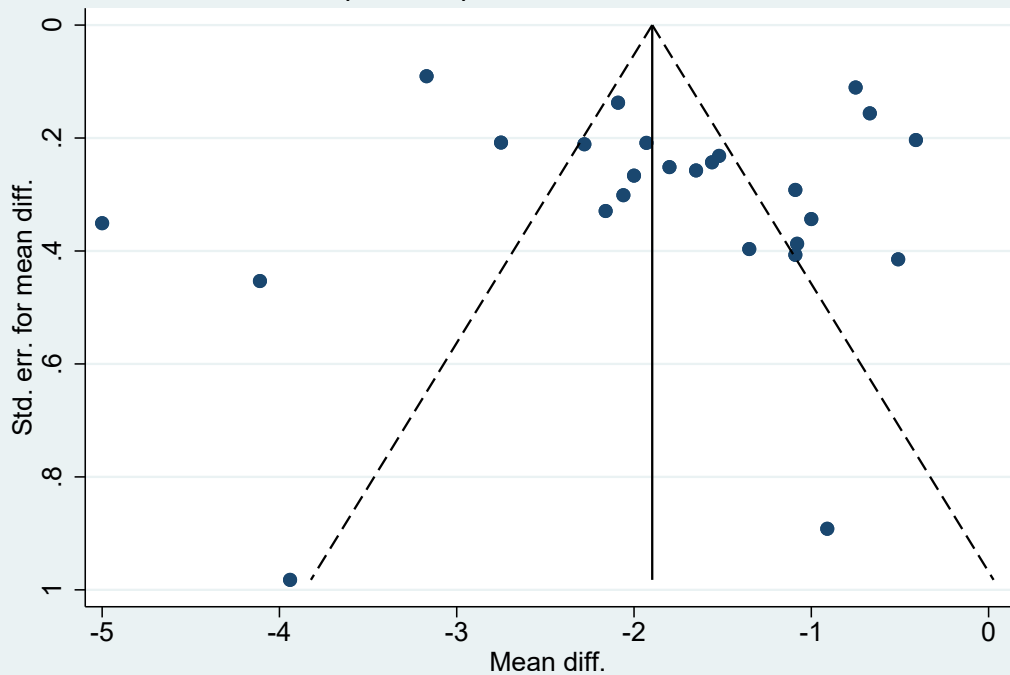

Supplement: Supplementary file 1 [file Data_Sheet_1.zip › Add supplementary charts/Supplementary Figure 1.pdf]

Funnel plot with pseudo 95% confidence limits

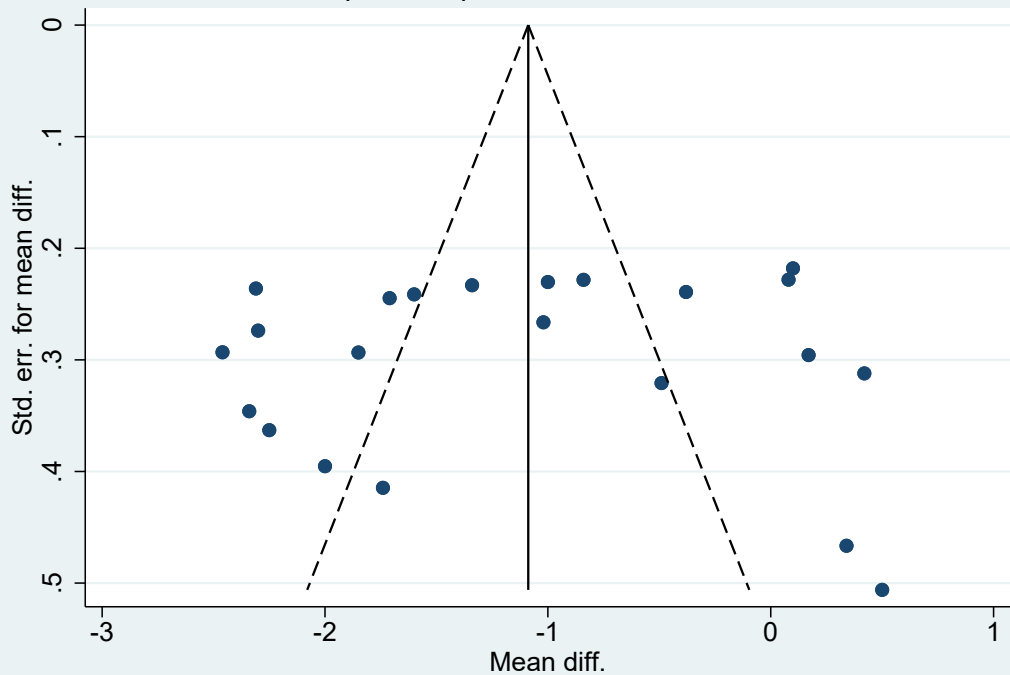

Supplement: Supplementary file 1 [file Data_Sheet_1.zip › Add supplementary charts/Supplementary Figure 2.pdf]
